# Supplementary material for: Validating the Chinese geriatric trigger tool and analyzing adverse drug event associated risk factors in elderly Chinese patients: A retrospective review
Source: PLoS One. 2020 Apr 28;15(4):e0232095. doi: 10.1371/journal.pone.0232095 (PMC7188209; doi:10.1371/journal.pone.0232095)
Supplement: S1 Table — (DOCX) [file pone.0232095.s001.docx]

S1 Table: Initial Chinese geriatric trigger tool

| No. | Triggers | No. | Triggers |
| --- | --- | --- | --- |
| Laboratory index | | 22 | Vitamin K |
| 1 | PTT > 100s | 23 | Antiallergic |
| 2 | INR > 5 | 24 | Romazicon (Flumazenil) |
| 3 | Glucose < 2.8mmol/L | 25 | Naloxone (Narcan) |
| 4 | Rising BUN or serum creatinine greater than 2 times baseline | 26 | Anti-emetic |
| 5 | ALT (or AST) ≥3 ULN and / or ALP≥2 ULN and T-BIL > 2UNL (can have abnormal INR) | 27 | Antidiarrheal |
| 6 | PLT < 75×10^9^/L | 28 | Laxative |
| 7 | WBC < 3.0×10^9^/L | 29 | 50% glucose |
| 8 | HGB > 170g/L(man), > 150g/L(woman) | 30 | Protamine |
| 9 | Decrease of greater than 25% in hemoglobin or hematocrit | 31 | Epinephrine |
| 10 | K^+^ < 3.5mmol/L | 32 | Glucose injection and regular insulin |
| 11 | K^+^ > 5.5mmol/L | 33 | Transfusion or use of blood products |
| 12 | Ca^2+^ > 2.62 mmol/L | 34 | Insulin (regular insulin or insulin analogue) used in non-diabetics |
| 13 | TSH < 0.27 mU/L or FT4 > 22.40 pmol/L | Clinical symptoms | |
| 14 | TSH > 4.2mU/L or FT4 < 12.0 pmol/L | 35 | Over sedation/hypotension |
| 15 | Clostridium difficile positive | 36 | Rash |
| Plasma concentration | | 37 | Dehydration |
| 16 | Digoxin > 2 ng/ mL | 38 | Psychosis |
| 17 | Gentamicin or Tobramycin levels peak > 10mg/L, trough > 2mg/L | 39 | Heart rates <60/min |
| 18 | Cyclosporin > 300µg/mL | 40 | Respiratory rates < 12 /min |
| 19 | Theophylline > 20mg/L | Intervention | |
| 20 | Tacrolimus > 20 ng/mL | 41 | Abrupt medication stops |
| 21 | Voriconazole levels > 5.5mg/L | Other | |
| Antidotes | | 42 | Others ADEs (ADEs not related to one of the triggers listed above) |
