# Supplementary material for: Validating the Chinese geriatric trigger tool and analyzing adverse drug event associated risk factors in elderly Chinese patients: A retrospective review
Source: PLoS One. 2020 Apr 28;15(4):e0232095. doi: 10.1371/journal.pone.0232095 (PMC7188209; doi:10.1371/journal.pone.0232095)
Supplement: S2 Table — (DOCX) [file pone.0232095.s002.docx]

S1 Table: Proportion of diseases among older patients

| Diseases | n | % |
| --- | --- | --- |
| Neoplasms | 631 | 35.06% |
| Circulatory | 237 | 13.17% |
| Digestive | 186 | 10.33% |
| Respiratory | 149 | 8.28% |
| Visual | 144 | 8.00% |
| Neurologic | 132 | 7.33% |
| Musculoskeletal/ connective tissue | 103 | 5.72% |
| Genitourinary | 91 | 5.06% |
| Endocrine, nutritional or metabolic | 48 | 2.67% |
| Integumentary | 32 | 1.78% |
| Hematologic | 17 | 0.94% |
| Immunological | 13 | 0.72% |
| Mental, behavioral or neurodevelopmental | 13 | 0.72% |
| Ear or mastoid process | 4 | 0.22% |
| Total | 1800 | 100% |
