# Supplementary material for: Validating the Chinese geriatric trigger tool and analyzing adverse drug event associated risk factors in elderly Chinese patients: A retrospective review
Source: PLoS One. 2020 Apr 28;15(4):e0232095. doi: 10.1371/journal.pone.0232095 (PMC7188209; doi:10.1371/journal.pone.0232095)
Supplement: S3 Table — (DOCX) [file pone.0232095.s003.docx]

S3 Table: Multicollinearity diagnostic result

| Model | Unstandardized coefficients | | Standardized coefficients | t | *P* | Collinearity statistics | |
| --- | --- | --- | --- | --- | --- | --- | --- |
|  | B | Std. Error | Beta |  |  | Tolerance | VIF |
| (Constant) | 0.432 | 0.080 |  | 5.416 | 0.000 |  |  |
| Sex (Female) | 0.031 | 0.015 | 0.045 | 2.007 | 0.045 | 0.990 | 1.010 |
| Age | -0.004 | 0.001 | -0.095 | -4.229 | 0.000 | 0.976 | 1.024 |
| Length of stay | 0.006 | 0.001 | 0.165 | 4.697 | 0.000 | 0.401 | 2.496 |
| Number of medical diagnoses | 4.289×10^-5^ | 0.003 | 0.000 | 0.017 | 0.987 | 0.688 | 1.453 |
| Intensive care units | -0.113 | 0.017 | -0.167 | -6.616 | 0.000 | 0.768 | 1.303 |
| Number of admissions in the previous 1-year | 0.008 | 0.002 | 0.072 | 3.110 | 0.002 | 0.912 | 1.097 |
| Surgery | -0.110 | 0.017 | -0.158 | -6.580 | 0.000 | 0.849 | 1.177 |
| Method of admission | 0.023 | 0.022 | 0.027 | 1.056 | 0.291 | 0.730 | 1.370 |
| Treatment outcome | -0.008 | 0.024 | -0.008 | -0.336 | 0.737 | 0.920 | 1.087 |
| Drugs per patient | 0.006 | 0.002 | 0.110 | 2.966 | 0.003 | 0.359 | 2.786 |
| Doses per patient | -5.057×10^-5^ | 0.000 | -0.021 | -0.467 | 0.640 | 0.240 | 4.174 |
| Chinese patent medicine use | -0.001 | 0.024 | -0.001 | -0.049 | 0.961 | 0.918 | 1.090 |
