# Supplementary material for: Validating the Chinese geriatric trigger tool and analyzing adverse drug event associated risk factors in elderly Chinese patients: A retrospective review
Source: PLoS One. 2020 Apr 28;15(4):e0232095. doi: 10.1371/journal.pone.0232095 (PMC7188209; doi:10.1371/journal.pone.0232095)
Supplement: S4 Table — (DOCX) [file pone.0232095.s004.docx]

S4 Table: The complete result of stepwise logistic regression

| Variables | | B | SE | Wald | *P* | Exp(B) | 95%CI |
| --- | --- | --- | --- | --- | --- | --- | --- |
|  |  |  |  |  |  |  |  |
| Step 1 | Sex (Female) | 0.301 | 0.156 | 3.689 | 0.055 | 1.351 | 0.994-1.835 |
|  | Age | -0.045 | 0.010 | 20.125 | 0.000 | 0.956 | 0.937-0.975 |
|  | Length of stay | 0.045 | 0.009 | 25.627 | 0.000 | 1.046 | 1.028-1.065 |
|  | Number of medical diagnoses | -0.028 | 0.026 | 1.196 | 0.274 | 0.972 | 0.925-1.022 |
|  | Intensive care units | 0.655 | 0.380 | 2.967 | 0.085 | 1.925 | 0.914-4.054 |
|  | Number of admissions in the previous 1-year | 0.069 | 0.018 | 14.476 | 0.000 | 1.072 | 1.034-1.111 |
|  | Surgery | -1.291 | 0.210 | 37.833 | 0.000 | 0.275 | 0.182-0.415 |
|  | Method of admission (On foot) | -0.348 | 0.211 | 2.733 | 0.098 | 0.706 | 0.467-1.067 |
|  | Treatment outcome (Improve or cured) | 0.003 | 0.214 | 0.000 | 0.988 | 1.003 | 0.659-1.526 |
|  | Drugs per patient | 0.051 | 0.016 | 9.940 | 0.002 | 1.052 | 1.019-1.086 |
|  | Chinese patent medicine use | 0.085 | 0.217 | 0.153 | 0.696 | 1.088 | 0.712-1.664 |
|  | (Constant) | 0.449 | 0.687 | 0.427 | 0.514 | 1.566 |  |
| Step 2 | Sex (Female) | 0.301 | 0.156 | 3.690 | 0.055 | 1.351 | 0.994-1.835 |
|  | Age | -0.045 | 0.010 | 20.151 | 0.000 | 0.956 | 0.937-0.975 |
|  | Length of stay | 0.045 | 0.009 | 25.635 | 0.000 | 1.046 | 1.028-1.065 |
|  | Number of medical diagnoses | -0.028 | 0.025 | 1.198 | 0.274 | 0.973 | 0.925-1.022 |
|  | Intensive care units | 0.655 | 0.378 | 2.997 | 0.083 | 1.926 | 0.917-4.043 |
|  | Number of admissions in the previous 1-year | 0.069 | 0.018 | 14.594 | 0.000 | 1.072 | 1.034-1.111 |
|  | Surgery | -1.291 | 0.208 | 38.361 | 0.000 | 0.275 | 0.183-0.414 |
|  | Method of admission (On foot) | -0.348 | 0.210 | 2.746 | 0.097 | 0.706 | 0.468-1.066 |
|  | Drugs per patient | 0.051 | 0.016 | 9.951 | 0.002 | 1.052 | 1.019-1.086 |
|  | Chinese patent medicine use | 0.085 | 0.216 | 0.153 | 0.696 | 1.088 | 0.712-1.664 |
|  | (Constant) | 0.449 | 0.685 | 0.430 | 0.512 | 1.567 |  |
| Step 3 | Sex (Female) | 0.300 | 0.156 | 3.670 | 0.055 | 1.349 | 0.993-1.834 |
|  | Age | -0.045 | 0.010 | 20.097 | 0.000 | 0.956 | 0.937-0.975 |
|  | Length of stay | 0.045 | 0.009 | 25.535 | 0.000 | 1.046 | 1.028-1.065 |
|  | Number of medical diagnoses | -0.028 | 0.025 | 1.180 | 0.277 | 0.973 | 0.925-1.022 |
|  | Intensive care units | 0.646 | 0.378 | 2.920 | 0.087 | 1.907 | 0.910-3.999 |
|  | Number of admissions in the previous 1-year | 0.069 | 0.018 | 14.487 | 0.000 | 1.072 | 1.034-1.110 |
|  | Surgery | -1.292 | 0.208 | 38.457 | 0.000 | .275 | 0.183-0.413 |
|  | Method of admission (On foot) | -0.354 | 0.210 | 2.844 | 0.092 | 0.702 | 0.465-1.059 |
|  | Drugs per patient | 0.052 | 0.016 | 10.896 | 0.001 | 1.054 | 1.021-1.087 |
|  | (Constant) | 0.451 | 0.685 | 0.433 | 0.511 | 1.570 |  |
| Step 4 | Sex (Female) | 0.293 | 0.156 | 3.518 | 0.061 | 1.340 | 0.987-1.820 |
|  | Age | 0.046 | 0.010 | 21.184 | 0.000 | 0.955 | 0.937-0.974 |
|  | Length of stay | 0.043 | 0.009 | 24.595 | 0.000 | 1.044 | 1.027-1.062 |
|  | Intensive care units | 0.647 | 0.378 | 2.934 | 0.087 | 1.910 | 0.911-4.006 |
|  | Number of admissions in the previous 1-year | 0.067 | 0.018 | 13.815 | 0.000 | 1.069 | 1.032-1.107 |
|  | Surgery | -1.252 | 0.205 | 37.301 | 0.000 | 0.286 | 0.191-0.427 |
|  | Method of admission (On foot) | -0.397 | 0.206 | 3.703 | 0.054 | 0.673 | 0.449-1.007 |
|  | Drugs per patient | 0.047 | 0.015 | 9.787 | 0.002 | 1.048 | 1.018-1.080 |
|  | (Constant) | 0.443 | 0.683 | 0.420 | 0.517 | 1.557 |  |
