# Supplementary material for: Validating the Chinese geriatric trigger tool and analyzing adverse drug event associated risk factors in elderly Chinese patients: A retrospective review
Source: PLoS One. 2020 Apr 28;15(4):e0232095. doi: 10.1371/journal.pone.0232095 (PMC7188209; doi:10.1371/journal.pone.0232095)
Supplement: Attachment — Submitted filename: rebuttal letter.docx [file pone.0232095.s006.docx]

Thank reviewers for their comments. Most of questions have been answered in the manuscript, please saw the Revised Manuscript. However, there some questions should been use the space provided to explain. These answers are in the following.

1) Reviewer #1:

-Abstract: Conclusion: Why a shorter upgraded version?

Answer: According to the previous practice and expert opinion, some new triggers had been added to the initial list. We thought these triggers could cover most of ADEs. We thought about adding new trigger that were common in the study. The efficiency of the new triggers was inaccessible, because the denominators are unknow and the PPV of new triggers could not been obtain. In addition, too much triggers are not a good thing, the PPV of the tool may low to led to decrease efficiency. Therefore, the aim was shortening the trigger tool in this study.

-Is the sample used in this study considered as part of the presented data in the published article?

Answer: No. The ample used in this study is bigger than published article (25 per half month in this study; 20 per half month in published article), so we conducted a re-sampling with computer.

2) Reviewer #2:

-Line 123~124: Why did the authors choose significance level P<0.1, instead of P<0.05?

Answer: Because the interplay of variables, the results of univariate analysis were different to the multivariate analysis. Therefore, in general choosing significance level *P*<0.1 or *P*<0.15 was to avoid missing some important risk factors.

-3. Line 81~83, 99~100; Table 1, 3~5: Please make a single table showing the trigger, unadjusted odds ratio (OR) with 95% confidence interval (CI) and P-value, adjusted odds ratio (aOR) with the same attributes, and positive predictive value (PPV) of each trigger. The OR is taken from a univariate logistic regression for each trigger while the aOR is taken from multivariate logistic regression including each trigger with variables of patient characteristics. There were a lot of significant differences in patient characteristics between patients with and without ADEs, while those were not variables of interest. These may affect association between each trigger and the outcome. Since this study aim to reduce the triggers from the original tools, only triggers with significant aOR should be included for the final model. Then both original and upgraded trigger tools should be fitted using the same configuration of multivariate logistic regression. The patient characteristics should be forced into the models either those using original or upgraded triggers. No predictor selection should be applied for the model using original triggers. This feature/predictor selection method may or may not be applied to the model using upgraded triggers. However, it is suggested to use the selection method if the authors want to reduce the triggers even more. It is also suggested to compare selection method (forward vs. backward vs. stepwise) since stepwise selection do not always give the best prediction model compared to other selection method. In the end, the PPV using all triggers can be calculated from the predicted outcomes of the models using either original or upgraded triggers. Meanwhile, the PPV should be calculated from univariate logistic regression including one trigger and patient characteristics without any selection method.

Answer: The unadjusted odds ratio (OR) with 95% confidence interval (CI) and P-value have been showed in Table 1; positive predictive value (PPV) of each trigger have been showed in Tables 5 and 6.

These triggers are derived from clinical logic to flag medical records, which alerts reviewers to initiate further in-depth investigations regarding the patient’s record to determine the presence or absence of an adverse event; for example, a trigger is a value of blood glucose lower than 28 mg/dL in a patient with oral antidiabetic or insulin, which may alert professionals to perform a more detailed record review for evidence that the patient has an associated hypoglycemia. This method can be used in practice to track and assess ADE rates. The triggers and risk factor were two different things. The characteristics of patients was the risk factor of ADEs, which could be used to the prediction of ADE. However, the triggers were just to use to detect, but not used to the prediction of ADE. The “Preventable ADEs” meant the ADEs that were preventable which judged by the research team, but not meant the trigger could predict ADE.

PPVs = number of identified ADEs/ number of times the trigger. Therefore, the PPV was just the indicators of evaluating suitability for trigger, but had nothing to do with patients with or without ADEs. So. the OR and aOR of PPV could also not be calculated, and the aOR could not the inclusion criteria of triggers. We also could not conduct logistic regression to PPV% of each trigger. Because the results of OR and logistic regression of triggers could not get, the selection trigger was based on a similar study and our previous study, the triggers that were found to have a rate higher than a preestablished PPV cutoff (5%) were retained in the final tool.

3) Reviewer #3:

-According to the author's description "stepwise logistic regression was performed for variables associated with diagnoses at a significance level of p < 0.1", there should be 13 variables (7 categorical variables and 6 continuous variables) in the initial model. It would be better to represent the process of stepwise logistic regression in the Appendix and tell the readers which variables were removed due to collinearity.

Answer: the “Admission in the previous 1-year” and “Number of admissions in the previous 1-year” were the same thing. So, there were 12 variables (6 categorical variables and 6 continuous variables) in the initial model. The complete results of stepwise logistic regression and collinearity were showed in S3 and S4 Tables

-It was not clear how the authors separate 42 triggers into Table 4 and Table 5, as well as what the definition of positive was.

Answer: The triggers which PPVs%＞5 were showed in Table 4 (now is Table 5), and these triggers were retained in the final tool. The triggers which PPVs＜5 were showed in Table 5 (now is Table 6)
